# Hypoxia-Mimetic CoCl_2_ Agent Enhances Pro-Angiogenic Activities in Ovine Amniotic Epithelial Cells-Derived Conditioned Medium

**DOI:** 10.3390/cells11030461

**Published:** 2022-01-28

**Authors:** Miriam Di Mattia, Annunziata Mauro, Simona Delle Monache, Fanny Pulcini, Valentina Russo, Paolo Berardinelli, Maria Rita Citeroni, Maura Turriani, Alessia Peserico, Barbara Barboni

**Affiliations:** 1 Faculty of Bioscience and Technology for Food, Agriculture and Environment, University of Teramo, 64100 Teramo, Italy; mdimattia@unite.it (M.D.M.); vrusso@unite.it (V.R.); pberardinelli@unite.it (P.B.); mrciteroni@unite.it (M.R.C.); mturriani@unite.it (M.T.); apeserico@unite.it (A.P.); bbarboni@unite.it (B.B.); 2 Department of Biotechnological and Applied Clinical Sciences, University of L’Aquila, 67100 L’Aquila, Italy; simona.dellemonache@univaq.it (S.D.M.); fanny.pulcini@graduate.univaq.it (F.P.); 3 StemTeCh Group, Via L. Polacchi 11, 66100 Chieti, Italy

**Keywords:** amniotic epithelial cells, hypoxia, CoCl_2_, HIF-1α, stemness, conditioned media, VEGF, angiogenesis, apoptosis

## Abstract

Amniotic epithelial stem cells (AECs) are largely studied for their pro-regenerative properties. However, it remains undetermined if low oxygen (O_2_) levels that AECs experience in vivo can be of value in maintaining their biological properties after isolation. To this aim, the present study has been designed to evaluate the effects of a hypoxia-mimetic agent, cobalt chloride (CoCl_2_), on AECs’ stemness and angiogenic activities. First, a CoCl_2_ dose-effect was performed to select the concentration able to induce hypoxia, through HIF-1α stabilization, without promoting any cytotoxicity effect assessed through the analysis of cell vitality, proliferation, and apoptotic-related events. Then, the identified CoCl_2_ dose was evaluated on the expression and angiogenic properties of AECs’ stemness markers (*OCT-4, NANOG, SOX-2*) by analysing VEGF expression, angiogenic chemokines’ profiles, and AEC-derived conditioned media activity through an in vitro angiogenic xeno-assay. Results demonstrated that AECs are sensitive to the cytotoxicity effects of CoCl_2_. The unique concentration leading to HIF-1α stabilization and nuclear translocation was 10 µM, preserving cell viability and proliferation up to 48 h. CoCl_2_ exposure did not modulate stemness markers in AECs while progressively decreasing VEGF expression. On the contrary, CoCl_2_ treatment promoted a significant short-term release of angiogenic chemokines in culture media (CM). The enrichment in bio-active factors was confirmed by the ability of CoCl_2_-derived CM to induce HUVEC growth and the cells’ organization in tubule-like structures. These findings demonstrate that an appropriate dose of CoCl_2_ can be adopted as a hypoxia-mimetic agent in AECs. The short-term, chemical-induced hypoxic condition can be targeted to enhance AECs’ pro-angiogenic properties by providing a novel approach for stem cell-free therapy protocols.

## 1. Introduction

Stem cell-based regenerative medicine represents one of the most relevant challenges of the modern biomedical sciences. In this context, amniotic-derived epithelial cells (AECs) have acquired a relevant role due to their promising regenerative capabilities [1,2,3,4,5,6,7,8]. Benefiting from their early embryonic origin, these placenta-derived stem cells express several embryonic markers, such as SSEA-3, SSEA-4, TRA-1-60, and TRA-1-81, and pluripotent genes (*OCT-4*, *SOX-2*, *NANOG* AND *TERT*) in a highly conserved manner [9,10,11], proving their great differentiation potential [12,13]. In addition, since the placenta is fundamental for maintaining physiological foetus–maternal tolerance during pregnancy, AECs display low innate immunogenicity and anti-inflammatory properties [7,12,13]. Other relevant biological advantages of AEC sources are the cells’ great availability and the lack of ethical problems for their use. Moreover, their low immunogenicity, combined with their innate immunomodulatory activity, [13] has allowed their safe use in allogenic and xenogeneic transplantation pre-clinical settings [14,15,16,17,18,19], and even in clinical trials [20,21]. Furthermore, several studies have demonstrated that both AEC and derived factors have pro-regenerative influences [18,22], mainly exerted through paracrine actions that modulate inflammatory and anti-fibrotic mechanisms [23,24], as well as an angiogenic effect associated with wound healing [16,25].

Under physiological conditions, placenta-derived stem cells reside in a low oxygen (O_2_) environment [26]; moreover, AECs belong to an avascular tissue. Nevertheless, the current validated protocols for AECs do not consider that the hypoxic environment, within which AECs reside in vivo, averages about 2–8% O_2_ in humans and most mammalian species [27], identified as a crucial O_2_ tension level for early embryonic and placental development in mammals [26,27].

In these O_2_-controlled in vivo environments, stem cells may maintain a selective advantage suitable for their biological roles. Indeed, O_2_ availability, triggering specific intracellular signalling, can modulate gene expression, affecting cell fate [28]. Cellular adaptive responses to hypoxia are mainly mediated by the transcription factor hypoxia-inducible factor-1 (HIF-1), which induces transcriptional activation of various genes, promoting angiogenesis, cell self-renewal, and survival. When cells are exposed to hypoxia, at the molecular level, a complex signalling cascade triggers HIF-1α, which orchestrates a complex transcriptional program [29]. More in detail, in well-oxygenated environments, HIF-1α sub-units are hydroxylated at conserved proline residues by Prolyl-Hydroxylases (PHDs) and, after this modification, marked by the von Hippel–Lindau protein (pVHL) complex for proteasomal degradation. When O_2_ availability decreases, PHD activity diminished, stabilizing HIF-1α protein, which can induce transcription of genes with adaptive functions [30].

Consistent literature demonstrates a positive correlation existing between low O_2_ environment and stemness retention [28], even if few data have been collected to date on AECs. In this context, previous works have reported that AECs under 20% O_2_ spontaneously reduce the pluripotent expression genes [10] and go into epithelial-to-mesenchymal transition (EMT) [31,32]. On the other hand, 2% O_2_ culture conditions positively affect AECs’ differentiation towards the tenogenic lineage, inducing the up-regulation of tenogenic markers and improving the formation of tendon-like structures [33]. However, no studies to date have demonstrated the existence of a clear correlation between in vitro O_2_ tension and AEC biology, even if in vitro hypoxia might potentially be exploited to preserve or enhance positive therapeutic cell features.

In cell cultures, hypoxic conditions can be induced by modulating O_2_ levels with a specific gas mixture linked to a hypoxic chamber or incubator or directly with a tri-gas incubator [28]. However, to avoid variability linked to low oxygen incubator use, an alternative approach is represented by chemical “hypoxic mimetic compounds” treatments. Among them, cobalt chloride (CoCl_2_) is one of the most widely used to stabilize HIF-1α, even in normoxic conditions [34]. Cobalt exerts hypoxic action by occupying the iron-binding site on the PHDs, thus inhibiting the interaction between pVHL and hydroxylated HIF-1α. Using such a mechanism, CoCl_2_ artificially induces hypoxia by blocking HIF-1α degradation and favouring protein accumulation [35]. The stabilization of HIF-1α is essential to modulate key genes, such as VEGF, orchestrating neo-vascularization [36], thus generating pro-regenerative mechanisms that have already been exploited for therapeutic purposes [37]. In addition, it has been demonstrated that CoCl_2_ can increase the expression of stem cell markers (*Oct-4*, *Nanog*, *Sox-2*, and *c-Myc*) in a dose-dependent manner in cells collected from human exfoliated deciduous teeth [38] and dental pulp [39]. To the best of our knowledge, there are no other investigations about the effect of CoCl_2_ on promoting hypoxia in AECs.

Starting from this premise, since hypoxia conditions can preserve or enhance positive therapeutic cells features, the present study has been designed to study the effect of CoCl_2_-induced hypoxia on AECs. To this aim, the first step addressed defining the CoCl_2_ dose able to promote an acute hypoxia-mimetic effect without inducing cytotoxicity [40].

After this, the intra-cellular hypoxia-induced signalling was investigated by assessing the HIF-1α pathway and its major targets, VEGF, and the early and late signs of apoptosis were considered. Indeed, since AECs displayed a great regenerative potential [7] and HIF-1α has been widely linked to stemness retention and angiogenic induction, we hypothesized that its activation in AECs could preserve their stemness properties and enhance their angiogenic potential.

To confirm our hypothesis, the biological effect promoted by CoCl_2_ on AECs was investigated (i) by assessing their stemness markers expression and intracellular localization and (ii) by defining their pro-angiogenic activity through either the detection of released angiogenic factors or the ability to improve the in vitro angiogenesis.

The novelty of the presented data in this research demonstrates that 10 µM CoCl_2_ low dose activates the HIF pathway, thus mimicking a hypoxic environment in an ovine AECs (oAECs) culture. The in vitro hypoxic conditions maintain stem cell marker expression and enhance the naïve pro-angiogenic properties of the oAECs. Moreover, for the first time, it is demonstrated that oAEC-conditioned media are enriched with angiogenic factors that stimulate angiogenesis, even supporting the regenerative potential of this source of stem cells. Finally, oAEC-conditioned media (CM) could represent a suitable novel approach to be used for stem cell-free bio-therapy in regenerative medicine.

## 2. Materials and Methods

### 2.1. Ethics Issues

No ethic statement is required for the present research since the primary oAECs were collected from sheep slaughtered for feed purposes. Human umbilical vein endothelial cells (HUVECs) were purchased from Lonza (Walkersville, MD, USA).

### 2.2. oAECs Culture

The amniotic membranes were isolated from sheep uteri collected at a local slaughterhouse. To this aim, Appenninica sheep at the middle gestational stage determined by foetus dimension (ranging from 20 to 30 cm length) were considered. First, the oAECs were isolated from the amniotic membrane, as previously described [41]. Afterwards, freshly isolated oAECs were seeded in Petri dishes at the final concentration of 20.000 cells/mL in growth medium (GM) composed of alpha Eagle’s minimum essential medium (α-MEM, Gibco) supplemented with 10% FCS, 1% Ultra-Glutamine (Lonza), 100 U/mL penicillin (Lonza), 1% streptomycin (Lonza), and 1% amphotericin (Euro-lone). The cells were then incubated at 38.5 °C in 5% CO_2_ up to 70% of confluence.

### 2.3. Experimental Plan Designed to Study the Effect of Chemical-Induced Hypoxia on oAECs

First, a dose-effect experiment was designed to identify the concentration of Cobalt (II) chloride hexahydrate (CoCl_2_) (Sigma C2644, Sigma-Aldrich, St. Louis, MO, USA) necessary to induce a short-term hypoxic effect [42]. To this aim, oAECs were exposed to increasing doses of CoCl_2_: 0 (CTR), 10 µM, 50 µM, 100 µM, and 200 µM for 24 h and 48 h of culture.

The effects of CoCl_2_ were analysed by considering cell viability and proliferation, DNA integrity, and HIF activation at gene and protein levels.

Then, oAECs exposed to selected doses of CoCl_2_ for 1.5 h, 3 h, or 6 h were collected after 24 and 48 h of culture in serum-free media. Both cell extracts and conditioned media (CM) were collected at the end of the culture. More specifically, CMs were centrifuged to remove any cell debris and cryopreserved at −80 °C up until the analysis for pro-angiogenetic molecules contents and activity were assessed using the in vitro HUVEC proliferation and tubule formation assays. In addition, cells extracts were used to investigate the HIF-1α, early pro- and anti-apoptotic markers, stemness genes, and antigens, as well as VEGF protein expression.

**Figure 1:**
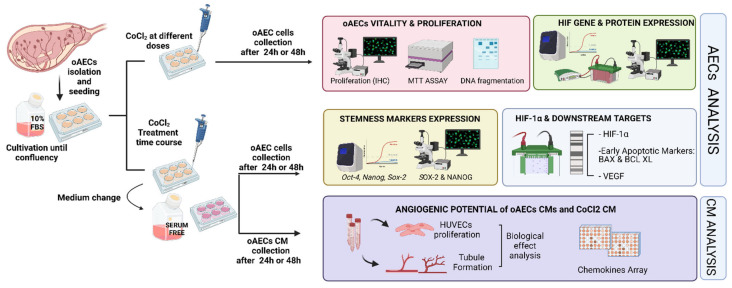
Experimental design. After oAECs isolation and culture until confluency, cells were treated or not with different CoCl_2_ concentrations for 24 and 48 h. Analysis on cell vitality and proliferation was firstly conducted to identify the optimal dose for further investigations. Then, in cells treated with CoCl_2_ concentrations at various time points, gene and protein expression of HIF-1α and its downstream targets were analysed. Finally, angiogenic assays investigations were performed on collected conditioned media (CM).

The experimental plan of the study is summarized in Figure 1.

### 2.4. CoCl_2_ Influence on Cell Viability and Proliferation

The oAECs exposed to dose-dependent concentrations of CoCl_2_ (0, 10 µM, 50 µM, 100 µM, and 200 µM) were analysed for:

1. MTT Assay: MTT assay (M5655-1G, Sigma-Aldrich, St. Louis, MO, USA) was performed to evaluate the effect of different CoCl_2_ doses on cell viability, according to the manufacturer’s instructions. Briefly, oAECs were seeded into 96-well plates (0.2 × 10^5^ cells/well) until reaching 70% confluence. After 24 h or 48 h from CoCl_2_ treatments, 20 µL MTT (5 mg/mL in PBS) was added to each well/sample and incubated at 37 °C for 3.5 h. Afterwards, the formazan crystals were dissolved in DMSO. Absorbance was measured at 595 nm with a micro-plate reader (Multi-mode Plate Reader EnSpire, Perkin Elmer). For each sample, the relative blank values were subtracted (i.e., the culture medium without cells). Data viability was normalized for the controls and expressed in percentage [23,31].
2. DNA Fragmentation: to exclude any toxic effect of CoCl_2_ on oAECs, the degrees of apoptosis induced at 24 and 48 h after treatments were evaluated. The analysis of DNA fragmentation was performed according to Kasibhatla et al. [43]. The detection of DNA fragments was conducted via agarose gel electrophoresis. After DNA extraction [43], 5 μg of each sample was suspended in 2 µL of sample buffer (0.25% bromophenol blue, 30% glycerol) and loaded into 1.8% agarose gel containing 1 µg/mL of GelRed (Bioline, Thomas Scientific, Swedesboro, NJ, USA) and run at a constant 70 V current. DNA fragments were visualized by an ultra-violet trans-illuminator (E-Gel Imager, Life Technologies, Thermo Fisher Scientific, Waltham, MA, USA).

### 2.5. Influence of Chemical-Induced Hypoxia on oAEC Genes’ Expression

Total RNA from oAECs untreated (CTR) and treated with different doses of CoCl_2_ were extracted with TriReagent (Sigma-Aldrich, St. Louis, MO, USA), according to our previous reports [23,31,33]. Quantification of total RNA samples was assessed using Thermo Scientific NanoDrop 2000c UV-Vis spectrophotometer at 260 nm. One μg of total RNA of each sample was used to synthesize the cDNA by Tetro Reverse Transcriptase (Bioline, Thomas Scientific, Swedesboro, NJ, USA) at a final volume of 20 Μl. Real-time Qpcr analysis for *HIF-1α* [33] and stemness *(OCT-4, NANOG, SOX-2*) [23,31] genes’ expression was performed by using primers sequences, shown in Table 1, and SensiFAST™ SYBR Lo-ROX kit (Bioline, Thomas Scientific, Swedesboro, NJ, USA). The reactions were carried out with 7500 Fast Real-Time PCR System (Life Technologies) by applying the two-step-cycling protocol for 40 cycles (10 s at 95 °C for denaturation and 30 s at 60 °C for annealing/extension) followed by melt-profile analysis (Applied Biosystems Real-Time PCR System, 7500 Software v2.3, Thermo Fisher Scientific, Waltham, MA, USA).

For each analysed gene, each sample was performed in triplicate, and values were normalized to the endogenous reference gene GAPDH. The relative expression of different amplicons was calculated by the comparative Ct (ΔΔCt) method and converted to relative expression ratio (2^−ΔΔCt^) [44] compared against untreated oAECs samples used as a control (CTR).

### 2.6. Analyses of Protein Profile Promoted by oAECs after Exposure to CoCl_2_

- Immunohistochemistry (IHC).

The analysis was performed on CTR- and CoCl_2_-treated oAECs to evaluate:

- proliferation by detecting the expression of nuclear marker KI-67 [4];
- stemness by detecting SOX-2 and NANOG protein cell localization [13];
- hypoxia-mediated signal by intra-cellular HIF-1α localization [45].

After 24 h or 48 h of culture, oAECs were fixed in 4% paraformaldehyde for 10 min, washed in PBS, permeabilized with 0.01% Triton-X in PBS, incubated with 2% (w/v) BSA in PBS for 1h at room temperature (RT), and then incubated with mouse anti-KI-67, rabbit anti-SOX-2 and rabbit anti-NANOG and mouse anti-HIF-1α, antibodies (Table 2) in 1% (w/v) BSA/PBS, overnight at 4 °C. The secondary antibodies (Table 2) were used for 1h at room temperature, in a dark chamber, for antigen retrieval (Table 2). Nuclear counterstaining was obtained with 4′,6-diamidino-2-phenylindole (DAPI, VECTASTAIN) at a final dilution of 1:5000 in PBS. The negative controls of each reaction were performed by omitting primary antibodies. The quantification of the number of cells expressing KI-67, SOX-2, and NANOG analysed proteins was determined by counting positive cells/100 cells counterstained with DAPI and expressed as a percentage of positive.

For image acquisition, an Axioskop 2 Plus incident light fluorescence microscope equipped with a CCD camera (Axioskop 2 Plus, Carl Zeiss, Oberkochen, Germany) possessing a resolution of 1300 × 1030 pixels and interfaced to a computer workstation provided with an interactive and automatic image analyser (AxioVision, Version 3.1, Carl Zeiss, Oberkochen, Germany) was used.

b. Western blot (WB) analysis.

According to previous works [33,46], WB detection was carried out to have a semi-quantitative value of the major protein target of intra-cellular cell response to hypoxia: HIF-1α, VEGF, and Bax and Bcl XL early apoptotic markers using specific antibodies (Table 2). Total proteins were extracted from CTR- and CoCl_2_-treated cells for 24 h and 48 h in lysis buffer (50 mM Tris HCl pH 8, 250 mM NaCl, 5 mM MEDTA, 0.1% Triton X-100 10%) with phosphatase inhibitor (P5726, Sigma-Aldrich, St. Louis, MO, USA) and protease inhibitor cocktails (P8340, Sigma-Aldrich, St. Louis, MO, USA). Cell extracts of samples were put on ice for 30 min and centrifuged at 12,000× *g* for 10 min at 4 °C, and then protein concentration was determined by using Quick Start™ Bradford 1x Dye Reagent (Bio-Rad Laboratories, Hercules, CA, USA. Subsequently, 35 µg of total proteins was separated in a 10% w/v acrylamide SDS–PAGE in TGS 1X running buffer (Bio-Rad Laboratories, Hercules, CA, USA) and then transferred onto nitrocellulose membranes (Millipore, Bedford, MA, USA) with TURBO Transfer (Bio-Rad Laboratories, Hercules, CA, USA).

Membranes were incubated with a blocking solution composed of 5% not fat-dried milk (Sigma-Aldrich, St. Louis, MO, USA) in 0.1% (v/v) Tween 20 in Tris HCl pH 7.5 (T-TBS) buffer saline for 1 h at room temperature. Specific primary antibodies against HIF-1α, Bax, Bcl XL, VEGF, and α-Tubulin (Table 2) diluted in TBS were incubated overnight at 4 °C.

Finally, the membranes were incubated with horseradish peroxidase (HRP)–conjugated anti-mouse or anti-rabbit secondary antibody (Table 2) for 1 h at RT. Target proteins were visualized by ECL substrate (LiteAblot PLUS, Euroclone, Milan, Italy). Chemiluminescent signal was detected with Azure’s 400 (Azure Biosystems, c400, Sierra Ct, Dublin, CA, USA).

Densitometric analysis was performed with Image J blot analyser software (ImageJ 1.53k, NIH, Bethesda, MD, USA). In detail, the protein bands were selected to assess their intensity. ImageJ program then displayed pictures of histograms, which showed each band’s intensity and the numerical value representing the band’s intensity normalized in a Tubulin expression [47].

Moreover, the VEGF, TGFβ-1, and TIMP-1 protein content in samples’ CM were also evaluated by WB. In detail, the proteins contained in 30 µL of undiluted CM, derived from untreated (oAECs CM) and 10 µM CoCl_2_ for 6 h-treated cells (CoCl_2_ CM) and kept without serum for 24 h, were separated in a gradient 4–15% w/v acrylamide SDS–PAGE in TGS 1X running buffer and then transferred onto nitrocellulose membranes. After the blocking solution step, the membranes were incubated with primary antibodies against VEGF, TGF-β1, and TIMP-1 (Table 2), followed by the incubation with the related secondary antibodies (Table 2). Target proteins’ chemiluminescent signals were detected, as reported above. Densitometric analyses of each band’s intensity was performed, and data were expressed as fold changes versus untreated oAECs CM values.

### 2.7. Pro-Angiogenic Influence of CM Derived from Naïve and Hypoxia-Induced oAECs

Xeno-biological tests of angiogenesis. Angiogenic tests were conducted using HUVECs to assess the pro-active influence of CM derived from CTR (naïve releasing activity) and CoCl_2_-treated oAECs (hypoxic releasing activity). To this aim, the following assays were performed:

a. Angiogenic protein markers’ array.

The membrane array developed for detecting the major pro-angiogenetic factors (Human Angiogenesis Ab Array-Membrane, Abcam, ab134000) [48] was used according to the manufacturer’s instructions. The membrane can simultaneously detect 20 different factors, including angiogenin, EGF, IL-8, VEGF-A, and VEGF-D. Briefly, the array membranes were incubated with a blocking buffer for 1 h at RT. Then, 1ml of oAECs CM and 10 µM CoCl_2_ for 6 h-treated cells (CoCl_2_ CM), all kept without serum for 24 h, were added to the membranes and incubated overnight at 4 °C on a rocking platform. Media α-MEM alone was used as a negative control.

Following washing steps, membranes were incubated with Biotinylated Antibody Cocktail (2 h) and then with HRP-Conjugated Streptavidin (2 h) at RT. The membranes were visualized using a chemiluminescent detection system (Euroclone, Milan, Italy). Chemiluminescent signal was detected with Azure’s 400 (Azure Biosystems, c400, Sierra Ct, Dublin, CA, USA). Densitometric analysis was performed with Image J blot analyser software (ImageJ 1.53k, NIH, Bethesda, MD, USA). The pixel density of each spot was determined by densitometry, and the average background was subtracted. Next, the positive control signal on each membrane was used to normalize the signal density of individual spots. Densitometry of mean pixel density was used for the semi-quantitative comparison between samples.

b. HUVEC Proliferation Assay.

Preliminarily, experiments were performed to study the endothelial cells proliferation test [49]. HUVECs (5000 cells/well) on a 96-well plate were grown in Endothelial Growth Medium-2 (EGM-2: Lonza, cat LOCC3162) and serum-free α-MEM (cultural medium of AEC during conditioned media collection). The xenogenic cultures were carried out the next day by replacing EGM-2 medium with CM derived from serum-free oAECs cultures at different dilutions: undiluted, 1:1, or 1:4 CM dilution in EGM-2 (CM, 1:1 CM, and 1:4 CM). As a control, HUVECs were, in parallel, cultured in EGM-2, α-MEM, or α-MEM diluted 1:1 and 1:4 in EGM-2.

Then, the endothelial cells proliferation tests were adopted to compare CM with CoCl_2_-derived CM effects. To this aim, CM derived from AECs exposed to 10 µM CoCl_2_ were collected after 24 and 48 h and used at the selected dilution (CoCl_2_ CM).

HUVEC proliferation was evaluated by fixing cells with 4% paraformaldehyde for 10 min and staining with crystal violet (1%). Stained cells were solubilized using a solution containing 1% SDS and 50% methanol. Absorbance was measured at 595 nm with a micro-plate reader (Multimode Plate Reader EnSpire, Perkin Elmer, Waltham, MA).

c. In Vitro Tubule-Like Formation.

Once CM dilution was identified, the tests were carried out to confirm CM effects on HUVEC tubule formation [49] as described below. The tubule formation assay is an advanced biological test based on the ability of HUVEC to form tubule-like structures when seeded on a matrix consisting of a mixture of basement membrane components (Matrigel). The assay was carried out using the in vitro Matrigel assay kit (Chemicon, Millipore) following the manufacturer’s instructions. In detail, the tubule formation assay was performed by coating 15-well micro-slides (10 µL/well) of IBIDI (Munich, Germany) with Matrigel and seeding 15.000 HUVECs to each well after 30 min of Matrigel solidification at 37 °C. For experimental aim, the HUVECs were incubated in CM and CoCl_2_ CM in 5% CO_2_ under a humidified environment in the air up to 16 h. As a control, HUVECs were, in parallel, cultured in EGM-2, α-MEM, or α-MEM diluted in EGM-2. The degree of the angiogenic response was assessed using an inverted phase-contrast microscope (Nikon) by evaluating the branching index obtained by measuring the number of junctions formed per vessel area. For each well, tubule-like structures images were acquired and quantified using an Image J angiogenesis analysis software. Three independent experiments were performed for each treatment.

### 2.8. Statistical Analysis

All experimental design investigations were performed on three badges of oAECs obtained from the different foetuses (biological replicates *n* = 3). The data are expressed as mean ± SEM obtained from 3 experimental replicates for each AEC badge (experimental replicates = 3 for each treatment/time point). Statistical analysis was performed using Prism 6 (GraphPad). One-way ANOVA was used for multiple comparisons and performed on data sets. Values were considered statistically significant, at least for *p* ≤ 0.05.

## 3. Results

### 3.1. Doses Effect of CoCl_2_ Treatment on oAECs Properties

**Figure 2:**
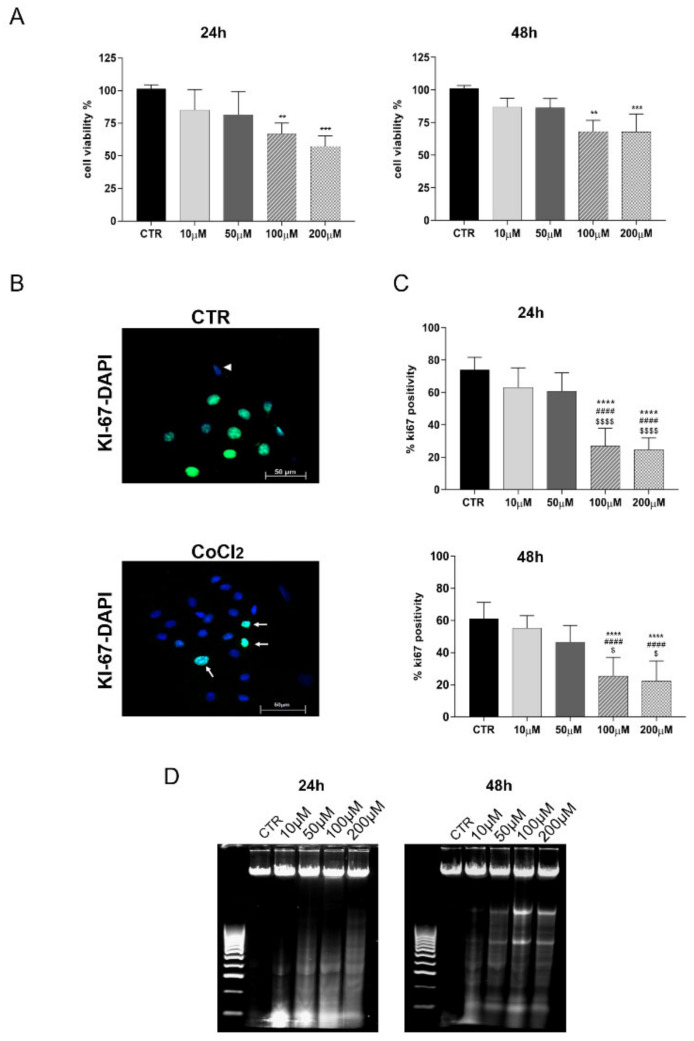
Dose-response assessment of CoCl_2_ on oAEC viability and proliferation. For this experiment, cells were treated with 0 (CTR), 10, 50, 100, and 200 µM of CoCl_2_ for 24 and 48 h of incubation. (**A**) The histograms show oAECs’ viability of untreated (CTR) and CoCl_2_-treated cells detected with the MTT assay. Data are expressed as a percentage of vital cells ± SEM obtained from experimental triplicates for each biological sample (*n* = 3 different animals). CTR viability was set as 100%. (**B**) Representative IHC images showing the nuclear localization of the proliferation marker KI-67 (green fluorescence) in oAECs cultured for 48 h in the absence (CTR, upper image) or presence of CoCl_2_ (lower image). Nuclei were counterstained with DAPI (blue fluorescence). Arrowheads indicate negative stemness nuclear localization in the cells, and arrows indicate positivity. Scale bar: 50 µm. (**C**) Histograms displaying the incidence of KI-67 positive cells recorded in CTR and CoCl_2_-treated oAECs at 24 and 48 h. The incidence value was expressed as a percentage of KI-67 positive cells/100 total cells ± SEM, obtained from triplicate experiments performed on three different biological samples. Values statistically different for ** *p* < 0.01, *** *p* < 0.001, **** *p* < 0.0001 vs. CTR, ^####^ *p* < 0.0001 vs. 10 µM CoCl_2_, and ^$^ *p* < 0.05, ^$$$$^ *p* < 0.0001 vs. 50 µM. (**D**) Representative agarose gel showing DNA fragmentation in CTR and CoCl_2_-exposed oAECs after 24 h and 48 h of culture.

MTT assay indicated that CoCl_2_ treatment affected cell viability in a dose-dependent manner (Figure 2). No toxic effects were observed in cells cultured for 24 h at doses lower than 50 µM (10 µM and 50 µM vs. CTR, both *p* > 0.05); on the contrary, cell viability dramatically decreased at 100 µM and 200 µM, respectively (100 µM vs. CTR, *p* < 0.05, and 200 µM vs. CTR, *p* < 0.001, respectively). CoCl_2_ exposure at lower doses did not modify cell proliferation when the incubation was extended up to 48 h (Figure 2A). The highest proliferative capacities of CTR and 10- or 50 µM-treated oAECs were confirmed at 24 h and 48 h through the assessment of KI-67: about 80% of cells were positive for the nuclear proliferative marker in both the treatments (Figure 2B,C). A significant drop in KI-67 positivity (<40%) was recorded in cells exposed to 100 µM (*p* < 0.0001 vs. CTR) and 200 µM (*p* < 0.0001 vs. CTR) of CoCl_2_ at either 24 or 48 h (Figure 2C). Of note, 50 µM of CoCl_2_ treatment promoted a slight decrease in cell viability and proliferation, although this flexion was not significant.

Moreover, the higher doses of CoCl_2_ promoted DNA fragmentation (Figure 2D). Specifically, signs of apoptosis were already detectable in oAECs exposed at a concentration of CoCl_2_ ≥ 50 µM after 24 h of culture (Figure 2D). The incidence of DNA fragmentation became slightly evident at 48 h when the first signs of DNA fragmentation were detected in 50 µM-treated cells (Figure 2D).

Based on dose-effect data, in the following experiments, concentrations higher than 50 µM of CoCl_2_ to promote in vitro chemical hypoxia were not considered due to their adverse effect on cell viability.

### 3.2. HIF-1α Pathway Is Activated by CoCl_2_-Treated oAECs

**Figure 3:**
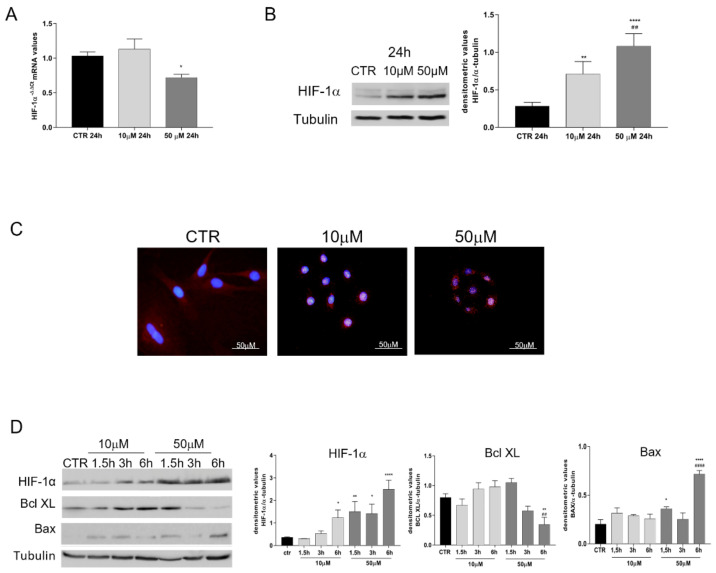
Evaluation of HIF-1α activation and early apoptotic markers in CTR and 10 and 50 µM CoCl_2_-treated oAECs. (**A**) RT-PCR analysis of HIF-1α at 24 h of CoCl_2_ incubation. Relative quantification of mRNA gene expression was calculated using the ΔΔCt method and presented as fold change to CTR (calibrator). (**B**) Representative Western Blot image of HIF-1α and relative densitometric values normalized on Tubulin expression. (**C**) Representative immunofluorescence images of HIF-1α localization (red fluorescence) in CTR and CoCl_2_-treated oAECs after 24 h of culture. Nuclei are counterstained with DAPI (blue fluorescence), Scale bar: 10 µm. (**D**) Time course of HIF-1α, Bcl XL, Bax proteins expression and relative densitometric values normalized on Tubulin expression. Data were expressed as mean ± SEM values of samples, each performed in triplicate, obtained at least from three different animals. Values statistically different for * *p* < 0.05, ** *p* < 0.001, **** *p* < 0.0001 vs. CTR, ^##^ *p* < 0.001, ^####^ *p* < 0.0001 vs. 10 µM CoCl_2_.

The activation of the HIF-1α pathway was analysed on oAECs cultured for 24 h without (CTR) or with 10 µM and 50 µM CoCl_2_ by combining Real-Time PCR, Western blot, and Immunofluorescence analyses (Figure 3).

*HIF-1α* gene expression (Figure 3A) was significantly down-regulated in oAECs exposed to 50 µM (Figure 3A, *p* < 0.05). In contrast, its protein stabilization was recorded in both groups of CoCl_2_-treated cells, even if the effect was significantly greater in 50 µM (*p* < 0.001 vs. 10 µM) (Figure 3B). The protein distribution performed using the IHC approach demonstrated a nuclear positivity for HIF-1α in the majority of cells belonging to both groups exposed to CoCl_2_, while no nuclear localization was observed in CTR cells (Figure 3C).

To study more in-depth the CoCl_2_ effect, the HIF-1α and early pro- and anti-apoptotic markers activation (Bax and Bcl XL, respectively) were evaluated in CoCl_2_ time course treatments. These experiments were designed by exposing oAECs for a short time (1.5 h, 3 h, and 6 h) to 10 µM and 50 µM CoCl_2_ (Figure 3D). A clear HIF-1α response was evident in 10 µM of CoCl_2_ exclusively after 6 h of culture (*p* < 0.05 vs. CTR), whereas 50 µM was able to promote a significant protein elevation starting from 1.5 h (*p* < 0.001 vs. CTR) up to 6 h (*p* < 0.0001 vs. CTR). However, AECs’ exposure to 50 µM CoCl_2_ led to the early activation of the pro-apoptotic marker (Figure 3D). Indeed, these oAECs displayed Bax on significantly higher levels than CTR after 1.5 h (*p* < 0.05 vs. CTR) and 6 h (*p* < 0.0001 vs. CTR and 10 µM CoCl_2_) of culture. The increase in Bax expression was accompanied, in parallel, by the down-regulation of the anti-apoptotic Bcl XL marker levels. Indeed, Bcl XL became significantly lower than CTR (*p* < 0.001, 50 µM CoCl_2_ vs. CTR) and 10 µM CoCl_2_ after 6 h of treatment (*p* < 0.05, 50 µM CoCl_2_ 6 h vs. 10 µM CoCl_2_). However, oAECs exposed to 10 µM CoCl_2_ did not show any significant variation over time in both Bax and Bcl XL proteins (*p* > 0.05, vs. CTR). These data confirmed the previous results of DNA fragmentation, demonstrating that 50 µM CoCl_2_ treatment induced a precocious activation of apoptosis in oAECs (Figure 2D). In contrast, the exposure of oAECs to 10 µM CoCl_2_ was able to activate the HIF-1α pathway without affecting apoptosis (Bax and Bcl XL both *p* > 0.05 vs. CTR).

**Figure 4:**
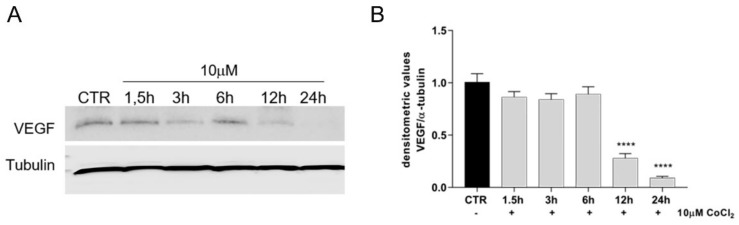
Analysed VEGF expression, in untreated oAECs (CTR) and 10 µM CoCl_2_-treated oAECs. (**A**) Representative Western blot image of VEGF expression in a time course experiment conducted at 1.5 h, 3 h, 6 h, 12 h, and 24 h. (**B**) Densitometric values of VEGF were normalized on Tubulin expression. Data were expressed as mean ± SEM values of samples, each performed in triplicate, obtained at least three different animals. Values statistically different for **** *p* < 0.0001 vs. CTR.

The activation of HIF-1α related pathways was also analysed by assessing the levels of VEGF, one of its major downstream targets (Figure 4). The 10 µM CoCl_2_ treatment maintained the intracellular expression of the VEGF at similar levels to freshly isolated cells (T0, data not shown) and/or oAECs CTR up to 6 h of incubation (*p* < 0.0001 vs. CTR), then VEGF levels significantly decreased at 12h, becoming undetectable after 24 h (*p* < 0.0001 vs. CTR).

### 3.3. CoCl_2_ Effects on Pluripotency Markers

**Figure 5:**
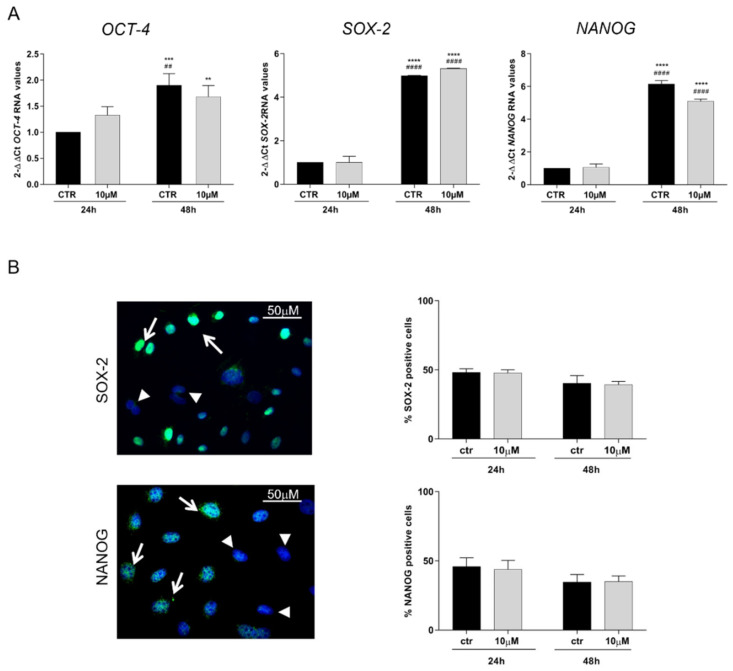
Evaluation of stemness markers expression in untreated (CTR) and 10 µM CoCl_2_-treated cells for 6 h, kept in culture for 24 h or 48 h. (**A**) Representative quantitative RT-PCR analysis of *OCT-4, SOX-2,* and *NANOG* stemness genes. Relative quantification of each target’s gene expression was calculated using the ΔΔCt method and presented as fold change to the CTR 24 h (calibrator). (**B**) Representative IHC images of SOX-2 and NANOG proteins nuclear localization (green fluorescence, arrows) in CTR and CoCl_2_-treated oAECs. Nuclei were counterstained with DAPI (blue fluorescence). Arrowheads indicate negative stemness nuclear localization in the cells. Scale bar: 10 µm. Histograms show the percentage of cells with positive nuclei to SOX-2 or NANOG/100 total cells. Values statistically different for ** *p* < 0.01, *** *p* < 0.001, **** *p* < 0.0001 vs. CTR 24 h and ^##^ *p* < 0.01, ^####^ *p* < 0.0001 vs. 10 µM 24 h.

To evaluate whether chemical hypoxia was able to modulate stemness properties of oAECs, the mRNA expression of pluripotency markers *OCT-4, NANOG,* and *SOX-2* was evaluated in untreated CTR and 10 µM CoCl_2_ for 6 h-treated cells and cultured after 24 h and 48 h (Figure 5). AECs up-regulated all the pluripotency genes at 48 h (Figure 5A, *p* < 0.05 CTR 48 h vs. CTR 24 h and 10 µM 24 h) and regardless of 10 µM CoCl_2_ treatment (*p* > 0.05, 10 µM CoCl_2_ vs. CTR both at 24 and 48 h). In contrast, the nuclear distribution of the NANOG and SOX-2 proteins did not show any time-dependent elevation neither in CTR nor in CoCl_2_-related cells (Figure 5B). Unfortunately, the analysis of OCT-4 intra-cellular distribution was not carried out due to the absence of a specific ovine antibody able to discriminate among the three main isoforms (OCT-4A, OCT-4B, and OCT4-B1).

### 3.4. Growth Factor Expression in CoCl_2_-Treated oAECs Conditioned Media

The ability of oAECs to release the angiogenic factors in response to chemical hypoxia was assessed by examining pro-angiogenetic factors released in CM. More specifically, the CM were collected 24 h after a 6 h-long oAECs pre-treatment with 10 µM CoCl_2_ (CoCl_2_ CM, i.e., oAECs hypoxic production) or without CoCl_2_ (oAECs CM, the oAECs naïve production). The 6h pre-treatment was selected in accordance with previous data reporting this as the best CoCl_2_-treating time able to stimulate VEGF and HIF-1α expression (Figure 3 and Figure 4).

**Figure 6:**
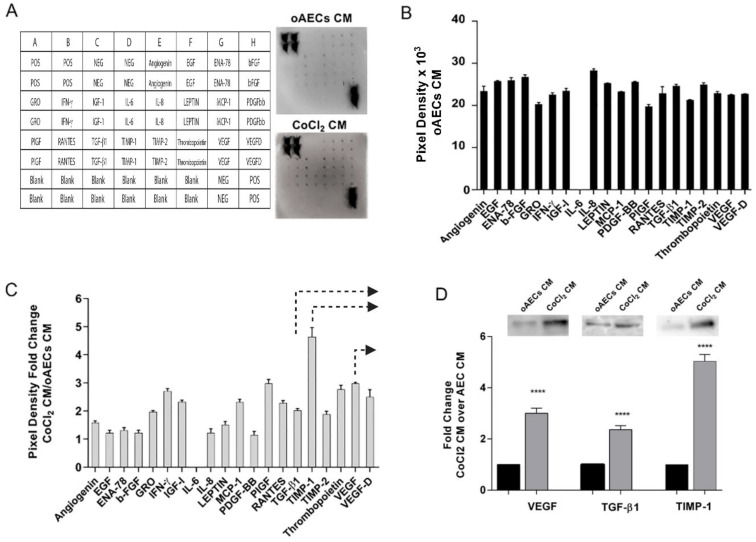
Angiogenic factors profile expression. (**A**) The layout of the array and representative images of chemokines detection. (**B**) Quantification by densitometric analysis of antibodies array of untreated oAECs CM, collected after 24 h after medium change. (**C**) Angiogenic proteins profile expression of CoCl_2_ CM. Data are expressed as fold changes versus untreated oAECs CM values. Arrows indicate the angiogenic factors further analysed in WB. (**D**) Representative WB images of VEGF, TGF-β1, and TIMP-1 protein content in untreated oAECs CM and CoCl_2_ CM, collected after 24 h after medium change. Densitometric values are expressed as fold changes versus untreated oAECs CM values. Values statistically different for **** *p* < 0.0001 vs. oAECs CM.

The CM analyses were performed by using a human cytokine antibody array made for 20 pro-angiogenic factors (Figure 6). The protein analysis showed that oAECs CM itself contains high levels of chemokines and angiogenic factors (Figure 6B). The exposure of oAECs to 10 µM CoCl_2_ led to an overall enhancement in all angiogenetic factors’ release. In particular, GRO, IFN-γ, IGF-I, MCP-1, PIGF, RANTES, TGF-β1, TIMP-1, TIMP-2, Thrombopoietin, VEGF, and VEGF-D levels were 2-fold or more higher compared to the corresponding target expression level in oAECs CM (Figure 6C). Moreover, the significant increased secretion of VEGF, TGF-β1, and TIMP-1 proteins in CoCl_2_ CM (all factors, *p* < 0.0001 CoCl_2_ CM vs. oAECs CM) were also detected in Western blot analyses, supporting cytokine array results (Figure 6D). In detail, VEGF, TFG-β1, and TIMP-1 protein content in CoCl_2_ CM were 3-fold, 2.4-fold and 5.2-fold higher, respectively, compared to the corresponding protein levels in oAECs CM (Figure 6D), similarly to those observed in the array data (Figure 6C).

### 3.5. Pro-Angiogenic Effect Induced by Chemical Hypoxia on AECs

The pro-angiogenic effect induced by chemical hypoxia was then biologically tested by exposing HUVEC culture to CM. To this aim, CM were collected after 24 or 48 h from AEC’s pre-treatment (6 h) with or without 10 µM CoCl_2_

**Figure 7:**
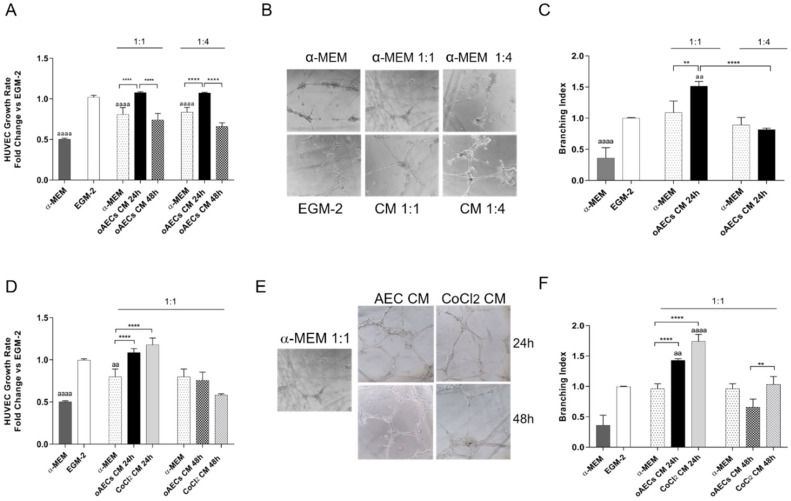
Evaluation of angiogenic potential of CM collected from untreated oAECs (oAECs CM) or 10 µM CoCl_2_-treated oAECs derived CM (CoCl_2_ CM). (**A**) HUVEC proliferation fold change of CM collected after 24 h or 48 h in culture was compared with those of endothelial cells cultured in EGM-2 (Endothelial Growth Medium) or different dilution of α-MEM (basal cultural medium of AEC): undiluted and 1:1 or 1:4 dilution in EGM-2. (**B**) Tubule formations images and (**C**) relative branching index values. (**D**) HUVEC proliferation in HUVECs exposed to CM or CoCl_2_ CM (selected 1:1 dilution) after 24 or 48 h. Values are expressed as fold changes vs. EGM-2. (**E**) Images of tubule formation with CM or CoCl_2_ CM and calculation of the branching index (**F**). Values statistically different for ** *p* < 0.01, **** *p* < 0.0001 vs. α-MEM in EGM-2, or CM ^aa^ *p* < 0.01, ^aaaa^ *p* < 0.0001 vs. EGM-2.

Since the trans-specificity of HUVECs’ angiogenic assays was never previously demonstrated, preliminary experiments were carried out to set up CM dilution to use in culture with human endothelial cells. First, the biological activities of ovine-derived CM were tested using undiluted CM (not shown) or 1:1 and 1:4 dilution in EGM-2 (Figure 7A). In order to exclude any angiogenic properties of α-MEM used for AECs culture, the HUVEC proliferation assay was also performed in α-MEM undiluted, as negative CTR, or diluted 1:1 or 1:4 in EGM-2 (Figure 7A).

Using this experimental setup, CMs were assayed for HUVEC growth response and tubule-like structures formation.

The lowest HUVEC proliferation was observed in cells incubated in α-MEM (*p* < 0.0001 vs. EGM-2, positive control). Analogously, the HUVEC growth was significantly compromised when cell cultures were performed in 1:1 and 1:4 diluted α-MEM (both dilutions, *p* < 0.0001 vs. EGM-2). As shown in Figure 7A, this trend was reversed by exposing HUVEC to CM collected after 24 h at both the dilutions (*p* < 0.0001 vs. 1:1 and 1:4 α-MEM dilution in EGM-2). Under this cultural condition, the HUVEC displayed a degree of proliferation similar to that recorded in undiluted EGM-2. A dramatic reduction of CM-inductive HUVEC proliferative effect was observed using CM collected at 48 h regardless of the dilution (for 1:1 and 1:4 dilutions *p* < 0.0001 vs. 24 h AECs CM, respectively, see Figure 7A). Based on these preliminary results, the experiment setup was moved towards the tubule-like structures formation assay by exposing the HUVEC exclusively to CM collected after 24 h of cultures (Figure 7B). As shown in Figure 7C, the analysis of branching index, the best parameter able to measure endothelial tubule formation ability, demonstrated that tubule-like structure formation was scarcely promoted in α-MEM (*p* < 0.05 vs. EGM-2 and vs. dilutions of α-MEM in EGM-2). The highest branching index was recorded in HUVEC exposed to CM diluted 1:1 in EGM-2 (*p* < 0.01 vs. EGM-2).

Once the trans-specific (xeno) conditions were set up as well as the CM dilution (1:1), both assays were used to compare the pro-angiogenic effect of CM vs. CoCl_2_ CM collected in 24 and 48 h. As shown in Figure 7D, CoCl_2_ CM did not enhance the naïve effect promoted by oAECs CM on HUVECs’ growth responses at 24 h. However, both CM were able to stimulate HUVEC growth rate over the values recorded in HUVECs cultured under control condition (both vs. α-MEM diluted 1:1 in EGM-2: *p* < 0.0001; Figure 7D).

On the contrary, the HUVECs’ culture on Matrigel showed that CoCl_2_ CM markedly enhanced tubule-like structure formation (Figure 7E): the branching index was 0.5-fold higher in HUVECs exposed to CoCl_2_ CM than to CM (*p* < 0.0001) (Figure 7F). In addition, the tubules like structures developed in CoCl_2_ CM-treated HUVECs displayed a different degree of maturity with a great number and thickness of branches. Again, the influence of CoCl_2_ CM on the branching index dropped at 48 h even if the tubule-like structure formation still resulted significantly higher than in HUVECs exposed to CM (*p* < 0.0001) (Figure 7F).

## 4. Discussion

The optimization of cell culture conditions represents a key challenge to preserve stem cell properties as well as to develop a cultural strategy to improve their function for regenerative medicine purposes. Even if several pieces of evidence converge in demonstrating that in vitro parameters can influence stem cell properties [50], the current stem cell expansion protocols are frequently set up without considering the native environmental conditions. An underestimated cultural parameter is the oxygen tension that in the majority of stem cell amplification protocols is maintained at air tension levels because it is forgotten that, instead, the natural niche stem cell environment recognizes physiologically low O_2_ tension in several districts [28].

Technologically, O_2_ in cell cultures can be modulated by using low oxygen pre-mixed tanks or a special tri-gas incubator [28]. Nevertheless, maintaining cell exposure to a constant condition of low O_2_ tension is not always guaranteed, even by working with a controlled oxygen incubator; thus, an alternative way to induce hypoxia is the use of mimetic chemical agents employed primarily for this purpose [40]. One of the most-used compounds is CoCl_2_•6H_2_O, proposed for its capacity of stabilizing HIF-1/2α even when cells are exposed to normoxic conditions [51]. CoCl_2_ artificially induces hypoxia-mediated signalling by blocking HIF degradation and leading to protein accumulation by maintaining cells in atmospheric air in the status of induced hypoxia also during the manipulation procedures [42].

However, since CoCl_2_ may exert a dramatic toxic effect on cells [34] when used at high doses, it can be used exclusively for designing short-term protocols and after setting up the correct dose inducing hypoxia that may change based on the cell model [28].

It is well-known that Co is an essential biological element that, however, at high concentrations, may exert potential adverse health effects [52]. For example, in vitro studies have shown that CoCl_2_•6H_2_O at not-ideal concentrations may be genotoxic, causing DNA damage, inhibition of strand break repair, and apoptosis [53]. On the other hand, once the dose of CoCl_2_ has been defined, it can artificially induce hypoxia with a high degree of efficiency by blocking HIF degradation and leading to nuclear protein accumulation. In general, HIF-1α stabilization events have been observed in other cellular models from two hours to a maximum at 12–48 h under 100 µM and 300 µM doses of exposure [34].

In oAECs, the adaptive response to hypoxia through HIF-1α stabilization [54] was confirmed to be a transitory and a fast event that may be promoted even at a low concentration, such as 10 µM. Indeed, HIF-1α reached its highest level within the first 6 h of CoCl_2_ exposure, to then decrease until 24 h and become undetectable at 48 h (data not shown). However, its declining expression profile is compatible with the fast proteasome degradation [55] that makes the HIF-1α half-life the shortest among proteins [56].

Protein stability is the most reliable and distinctive tract of HIF-1α pathway regulation, while the modulation of HIF-1α at mRNA levels was more difficult to detect. In oAECs, as in other cellular models [57], the sustained hypoxia can progressively decrease HIF-1α mRNA displaying the opposite trend compared to that of the codified protein. Furthermore, the activation of HIF-1α-mediated signalling can even be documented by the detection of the protein translocation into the nucleus [45,56] occurring, in coherence with literature, from 6 h to 12 h [58], up to 24 h [59,60] (reviewed in [61]). In our stem cell model, the nuclear translocation of HIF-1α fluorescent signal was recorded after exposure to 10 µM and 50 µM of CoCl_2_ up to 24 h, whereas a cytoplasmic positivity characterized the control cells.

However, the data collected in the present research confirmed that the identification of the CoCl_2_ dose able to induce hypoxia could not be deduced exclusively by assessing HIF intra-cellular signalling activation or using cells’ proliferation data as indicative of cell viability. Indeed, the present results demonstrate that the hypoxia-mimetic dose required has to be identified in combination with different assays to obtain information on cell functions and, among them, the analysis of early pro-apoptotic markers activation was the most consistent [52]. According to data collected by other groups, the obtained results confirmed the ability of high doses of CoCl_2_•6H_2_O in promoting several cell damages such as genotoxicity, DNA fragmentation, strand break repair, oxidative stress, and apoptosis that are worth to be assessed with great attention [53].

Since, in literature, no previous works on oAECs CoCl_2_ treatment were available, dose- and time-dependent experiments were designed in order to compare a low (10 µM) with an intermediate (50 µM) and two high concentrations (100 µM and 200 µM) for 24 or 48 h.

The MTT and KI-67 assays together demonstrated a dose-dependent negative impact of CoCl_2_ on oAEC proliferation that was, however, mainly ascribable to the higher concentrations, while a significant reduction of cell division was already visible after 24 h of culture.

The dose-dependent effect was better refined by the analysis of the apoptotic gel ladder that displayed that the incubations longer than 24 h were all able to trigger DNA fragmentation except in 10 µM and 50 µM oAEC groups. However, it should be noted that this late apoptosis event was not sufficiently sensitive to identify the pro-apoptotic role of 50 µM cell exposure, such as the profile analysis of pro- and anti-apoptotic early genes. Indeed, the mimicking hypoxia dose was finally identified by taking advantage of the inverse expression profile of Bcl XL and Bax during the first 6 h of treatment with 50 µM, indicating that the unique CoCl_2_ dose able to induce a hypoxia-mimetic effect and together preserve cell viability was 10 µM.

These results proved, indeed, that hypoxia per se can induce apoptosis and that the severity of this condition determines whether cells become apoptotic or adapt themselves to survive [62]. When brought together, this evidence led to the conclusion that 10 µM for 6 h represents the most suitable setup for oAECs to stabilize HIF-1α without affecting cell proliferation and without triggering any early or late apoptotic mechanisms.

Once stabilized, HIFs factors can induce the transcription of more than 70 genes correlated with O_2_ homeostasis, such as angiogenesis, mitochondrial metabolism, and adaptive functions related to different biological responses. The regulation of self-renewal properties by O_2_ can also depend on HIF stabilization directly through stemness genes modulation or indirectly via metabolic-related factors [63].

In this context, different reports indicate a positive correlation between HIF-1α stabilization after CoCl_2_ treatment and stemness markers expression [28].

However, no significant differences were recorded in the present research after 24 h or 48 h of culture in oAEC expression and nuclear location of the three major pluripotency transcription factors (*OCT-4*, *SOX-2*, and *NANOG*), thus suggesting that HIF activation does not impact cell stemness.

Hypoxic conditions, mediated by the HIF-1α stability and dimerization, are associated in several cellular models with the transcription of VEGF, a determinant chemokine for leading angiogenesis [64]. Surprisingly, VEGF protein did not increase after the CoCl_2_-treatment in oAECs. On the contrary, the major angiogenetic factor displayed a constant level during the first 6 h that then progressively decreased until it became almost undetectable at 24 h.

Nevertheless, the exclusive intra-cellular content of VEGF cannot be indicative of the pro-angiogenic activity of our cell model. Indeed, the analysis of the chemokines released in the conditioned media was more efficient in assessing the production of different chemokines and growth factors related to the angiogenic process. In this regard, our results demonstrate that different chemokines are produced by the cells, and their secretions are potentiated by the hypoxic condition CoCl_2_ mediated, evidencing a general increase for all the analysed factors. Moreover, as a support of the array results, the enhanced secretion of VEGF, TGF-β1, and TIMP-1 angiogenic factors was also observed by Western blot investigations in CM of cells after hypoxia conditioning. These results are in line with other studies reporting that hAECs secret considerable amounts of pro-angiogenic, anti-fibrotic, and anti-inflammatory factors, including bFGF, VEGF, angiogenin, and IGF, which represent all important bio-active secretory factors able to exert a therapeutic function in different disease models [65].

Among the factors, both VEGF-A and VEGF-D isoforms, the principal players in angiogenic activity, are highly secreted in CM, and their production is potentiated by CoCl_2_ exposure, suggesting that a hypoxic mimetic condition, via HIF activation, probably acts by promoting VEGF release [66,67] rather than an intra-cellular accumulation, also justifying the intra-cellular reduction previously reported by Western blot analysis.

Moreover, the placental growth factor (PlGF), a member of the VEGF family associated with placental angiogenesis [68], is highly up-regulated after the CoCl_2_ stimulus. In this context, it has been suggested that PlGF can heterodimerize with VEGF-A and acts synergistically to promote angiogenesis [69]. Furthermore, this result agrees with literature data reporting that PlGF levels can increase in association with elevated HIF-1α expression and that it competitively binds VEGF receptor 1, leading to an increase in VEGF-A availability, thus producing a stronger angiogenic stimulus [69]. Beyond HIF, even several cytokines factors can synergically influence VEGF up-regulation [68], including IGF-1, which plays a key role in stabilizing neo-vessel formations [70], or PDGF, the isoform of which, PDGF-CC, is determinant in highly active angiogenic tissues such as the placenta [71], both of which were released in our CM. In addition, secreted factors as TGF-β, VEGF, and IGF also exert substantial remodelling and anti-inflammatory effects through paracrine mechanisms, which play pivotal roles in stem cell-related repair mechanisms [72]. Even if some anti-angiogenic factors, such as TIMP-2, are up-regulated in the CM, further experiments are necessary to elucidate better the role of identified individual secreted factors by specific neutralization and/or silencing tests in single and/or combinatorial approaches.

Since the array analysis alone is not predictive of the cells’ pro-angiogenic activity, the biological tests on HUVEC proliferation and tubule-like structures formation have been determinant to assess the potential angiogenic effect of oAECs CM. This analysis allowed us to demonstrate firstly that our oAECs model can dialogue in a xeno-system by promoting HUVEC proliferation and tubule-like structures formation. Afterwards, the important naïve pro-angiogenic action of released factors in CM from untreated cells is demonstrated, supporting their angiogenic role when the cells are transplanted in tissue lesions favouring its regeneration process. In this context, it was observed that sinus explants, derived from sheep grafted with AECs, displayed an accelerated process of angiogenesis beyond a reduced fibrotic reaction and a limited inflammatory response [6]. Furthermore, in Achilles tendon injury, AECs establish an active dialogue with endogenous progenitor cells within the host tendons, inhibiting the inflammatory cell infiltration and accelerating blood vessel as well as extracellular matrix remodelling through the secretion of modulatory factors [18].

In particular, the CoCl_2_ hypoxia-mimetic compound potentiates the biological effect on HUVECs, especially on tubule-like structures formation, as confirmed by the branching index parameter. This is consistent with other cellular models [73] in which CoCl_2_-hypoxic induction, via HIF-1α activation, stimulated the secretion of angiogenic factors such as VEGF and angiogenin, which plays a critical role in neo-vascularization stabilizing the formation of the new tubule-like structures [66,67]. Our results are also supported by data on dental pulp stem cells (DPSCs), in which a comparative analysis of the cytokines and growth factors in the conditioned media from DPSCs and CoCl_2_-treated DPSCs showed that the growth factors associated with angiogenesis were higher in the secretory profile of CoCl_2_-treated cells [74]. Moreover, according to results reported on porcine and human MSCs, short-term exposure to hypoxia appeared superior to long-term cultures in augmenting the therapeutic characteristics of the cells [75]. Hence, the short-term chemical-induced hypoxic condition can be targeted to enhance oAECs’ pro-angiogenic properties.

In summary, the presented data confirm that hypoxic conditions enhance angiogenic effects of the AECs cellular model, that per se, exhibits a strong angiogenic potential.

## 5. Conclusions

CoCl_2_ can be used in oAECs culture to activate the HIF pathway, thus mimicking a hypoxic environment.

However, the analysis on cell viability revealed a great susceptibility of oAECs to high doses of CoCl_2_. In contrast, a low dose of 10 µM of CoCl_2_ was sufficient to promote HIF stabilization in oAECs without affecting cell proliferation and viability. Hypoxia can potentiate cell stemness features since it represents a more physiological condition for cell culture. However, the hypoxic stimulus with CoCl_2_ did not significantly improve stem cell markers’ expression; the levels remained similar to those of the control cells. However, it was found via HIF stabilization that hypoxic conditions enhanced cells’ naïve pro-angiogenic properties, positively affecting in vitro tubule-like structures formation. For the first time, the present research indicates that oAEC CMs are enriched with angiogenic factors that stimulate angiogenesis even in a xeno-system with HUVECs, supporting the regenerative potential of this source of stem cells. Moreover, AECs CM could represent a suitable novel approach for stem cell-free biotherapy in regenerative medicine.

## Figures and Tables

**Table 1:** Primers sequences details.

Gene	Forward Sequence	Reverse Sequence	References
***GAPDH***	5′-TCGGAGTGAACGGATTTGGC-3′	5′-CCGTTCTCTGCCTTGACTGT-3′	[23,31]
***HIF-1α***	5′-TGCTCATCAGTTGCCACTTC-3′	5′-TTTCCTCATGGTCACATGG-3′	[33]
***OCT-4***	5′-CTGCAGAAGTGGGTGGAGGAA-3′	5′-CTGCAGTGTGGGTTTCGGGCA-3′	[23,31]
***SOX-2***	5′-CACCCGCATGTACAACATGAT-3′	5′-TCTTAGGATTCTCTTGGGCCA-3	[23,31]
***NANOG***	5′-TGGATCTGCTTATTCAGGACAG-3′	5′-TGCTGGAGGCTGAGGTATTTC-3′	[23,31]

**Table 2:** Details of primary and secondary antibodies used.

**Antibodies Used for IHC Analysis**			
**Primary Antibody** **(Company Information)**	**Primary Antibody Dilution**	**Secondary Antibody** **(Company Information)**	**Secondary Antibody Dilution**
KI-67 (Santa Cruz, sc-101861)	1:50	Mouse Alexa Fluor 488 (Abcam, ab 150113)	1:100
HIF-1α (Novus Bio, 100-123)	1:100	Mouse CY Conjugated (Millipore, AP124C)	1:500
SOX-2 (Abcam, ab69893)	1:200	Rabbit Alexa Fluor (Abcam, ab 150077)	1:500
NANOG (Millipore, AB9220)	1:1000	Rabbit Alexa Fluor (Abcam, ab 150077)	1:500
**Antibodies Used for WB Analysis**			
HIF-1α (Novus Bio, 100-123)	1:500	Anti-mouse HRP conjugated (Santa Cruz, sc-516102)	1:10000
VEGF (Calbiochem, PC37)	1:500	Anti-rabbit HRP conjugated (Santa Cruz, sc-2357)	1:10000
Bax (Santa Cruz, sc 23959)	1:250	Anti-mouse HRP conjugated (Santa Cruz, sc-516102)	1:10000
Bcl XL (Santa Cruz, sc 8392)	1:250	Anti-mouse HRP conjugated (Santa Cruz, sc-516102)	1:10000
Tubulin (Sigma, T5168)	1:1000	Anti-mouse HRP conjugated (Santa Cruz, sc-516102)	1:10000
TGF-β1 (Abcam, ab27969)	1:250	Anti-mouse HRP conjugated (Santa Cruz, sc-516102)	1:10000
TIMP-1 (Millipore, AB770)	1:250	Anti-rabbit HRP conjugated (Santa Cruz, sc-2357)	1:10000

## Data Availability

The data supporting reported results can be available upon request to the authors.
